# A combination of SILAC and nucleotide acyl phosphate labelling reveals unexpected targets of the Rsk inhibitor BI-D1870

**DOI:** 10.1042/BSR20130094

**Published:** 2014-01-31

**Authors:** Alexander J. Edgar, Matthias Trost, Colin Watts, Rossana Zaru

**Affiliations:** *Division of Cell Signalling and Immunology, University of Dundee, Dow Street, Dundee DD1 5EH, U.K.; †MRC Protein Phosphorylation and Ubiquitylation Unit, College of Life Sciences, University of Dundee, Dow Street, Dundee DD1 5EH, U.K.

**Keywords:** BI-D1870, BIX 02565, dendritic cells, p90 ribosomal S6 kinase (Rsk), protein kinase B (PKB), Ste-20 like kinase, BMDC, bone marrow-derived dendritic cells, DC, dendritic cell, DMEM, Dulbecco’s modified Eagle’s medium, DSTT, Division of Signal Transduction Therapy, EGF, epidermal growth factor, ERK, extracellular-signal-regulated kinase, ERM, ezrin, radixin and moesin, FCS, foetal calf serum, GSK3, glycogen synthase kinase 3, IL6, interleukin 6, LPS, lipopolysaccharide, JNK, c-Jun N-terminal kinase, MAPK, mitogen-activated protein kinase, MIP1α, macrophage inflammatory protein 1α, PI3K, phosphoinositide-3 kinase, PIP3, phosphatidylinositol-3,4,5-triphosphate, PKB, protein kinase B, RSK, p90 ribosomal S6 kinase, SDC, spleen dendritic cells, SILAC, stable isotope labelling by amino acids in cell culture, TLR, Toll-like receptor, TNFα, tumour necrosis factor α

## Abstract

Protein kinase inhibitors frequently have interesting effects that cannot be fully ascribed to the intended target kinase(s) but identifying additional targets that might explain the effects is not straightforward. By comparing two different inhibitors of the Rsk (p90 ribosomal S6 kinase) kinases, we found that the increasingly used compound BI-D1870 had biological effects in murine DCs (dendritic cells) that could not be solely ascribed to Rsk or other documented targets. We assessed the ability of BI-D1870 and a second Rsk inhibitor, BIX 02565 to protect enzyme active sites from reaction with biotinylated nucleotide acyl phosphates. Using SILAC (stable isotope labelling by amino acids in cell culture)-labelled DC lysates as a source of enzyme targets, we identify several kinases that interact with BI-D1870 but not with BIX 02565. We confirmed that these kinases, including Slk, Lok and Mst1, are inhibited by BI-D1870 but to a much lesser extent by BIX 02565 and that phosphorylation of some of their substrates is blocked by BI-D1870 in living cells. Our results suggest that the BI-D1870 inhibitor should be used with caution. The SILAC-based methodology we used should be useful for further comparative unbiased profiling of the target spectrum of kinase inhibitors with interesting biological effects under conditions that closely mimic those found in cells.

## INTRODUCTION

The Rsk (90 kDa ribosomal S6 kinases) are Ser/Thr kinases that have been implicated in the regulation of a wide range of cellular functions such as cell proliferation and growth, apoptosis, metabolism and cell motility (reviewed in [1]). Rsks belong not only to the AGC kinase family but are also part of the MAPK (mitogen-activated protein kinase)-activated protein kinase group that are activated by the MAP kinases ERK (extracellular-signal-regulated kinase) 1/2 and/or p38 [2]. In mammals, four isoforms have been identified: Rsk1, 2, 3 and 4, Rsk1, 2 and 3 being the most abundantly expressed. They are composed of two functional kinase domains [3] that are sequentially activated by multiple phosphorylation steps. Briefly, upon activation, Erk1/2 mediates the phosphorylation of the C-terminal kinase [3], which in turn auto-phosphorylates a serine residue in the linker region [3,4] allowing the recruitment of PDK1 which, by phosphorylating the N-terminal kinase, leads to the activation of Rsk1, 2 and 3 [5,6]. Rsk4 appears to be constitutively active [7]. Owing to their involvement in the regulation of several key cellular processes it is not surprising that Rsks have been implicated in several pathological conditions. For instance, Rsk isoforms have been implicated in the development of some tumours [8–10]. Mutations in the *Rsk2* gene are thought to be responsible for the Coffin–Lowry syndrome that is characterized by skeletal malformations and severe psychomotor retardation [11]. These studies indicate that it is increasingly important to gain a better understanding of the specific roles of Rsk in different cell systems.

While the use of siRNAs, the overexpression of kinase dead Rsk mutants or cells from Rsk2-deficient mice or from patients with Coffin–Lowry syndrome have been helpful, the simultaneous expression of the Rsk1, 2 and 3 in most cell types has complicated the study of their cellular functions. To overcome this limitation, several inhibitors have been developed, which block either the Rsk N-terminal kinase such as SL0101 [12] and BI-D1870 [13] or the Rsk C-terminal kinase such as fmk (fluoromethylketone) [14]. Recently, another Rsk inhibitor, BIX02565 (hereafter BIX), has been described [15] which like BI-D1870 (hereafter D1870), acts as an ATP competitor. This cell-permeable compound was reported to be a highly specific RSK inhibitor with an *in vitro* IC_50_ of 1–2 nM. Notably, a screen against a panel of more than 200 kinases showed that only a few other kinases were affected and then only at ~20-fold higher levels of BIX.

So far, Rsk functions have been studied mainly in fibroblasts, neurons and osteoclasts (reviewed in [1]). Little is known about their role in the immune system and more particularly in DCs (dendritic cells). DCs are crucial players in the activation of the host defences against microbial pathogens [16]. Upon the recognition of pathogen-derived products such as LPS (lipopolysaccharide) by TLR (Toll-like receptors) a maturation programme is initiated, which includes the transient increase in antigen uptake and processing [17], the reorganization of the actin cytoskeleton and vacuolar compartments (reviewed in [18]) and the production of cytokines that will then lead to the activation of T cells [16]. Although a vast amount of work has been invested in the identification of the signalling pathways that regulate these processes, the detailed steps are still not fully understood. For instance, while the crucial role of Erk1/2 and p38 has been well documented, there is little information on which of their downstream kinase effectors are involved. We have previously shown that Rsk is activated in DC but, conversely to other cell types, not only by Erk1/2 but also by the p38 pathway via MK2/3 [19].

Here we have extended our dissection of the role Rsk plays in the regulation of DC functions downstream of TLR signalling using two different RSK inhibitors, D1870 and BIX. We show that although both compounds suppress cytokine production and antigen uptake by macropinocytosis in DC, they do so to strikingly different extents with D1870 having a significantly more potent effect. This aroused concerns about the specificity of this compound, which has now been used in multiple studies on Rsk [1,13,20]. We have used a kinase active site labelling system described by Patricelli et al. [21] and combined it with SILAC (stable isotope labelling by amino acids in cell culture)-based proteomic analysis of inhibitor specificity in DC lysates. We identify targets of D1870 other than Rsk including Slk, Mst1 and Lok all of which are members of the Ste20-like kinase family. We show that these kinases are constitutively active in DC and that their suppression by D1870 probably explains the effects of this inhibitor on some DC functions.

## MATERIALS AND METHODS

### Mice and cell culture

DCs were generated from the bone marrow or the spleen of C57BL/6 mice as previously described [17]. Briefly, bone marrow cells were cultured for 7 days at 37°C, 5% (v/v) CO_2_ in complete RPMI medium supplemented with 10 ng/ml recombinant granulocyte–macrophage colony-stimulating factor (GM-CSF; Peprotech). Spleen cells were cultured for 14 days at 37°C, 5% CO2 in complete RPMI containing 10 ng/ml GM-CSF and 1 ng/ml TGFβ (transforming growth factor-β; R&D Systems). NIH3T3 cells (European Cell Culture Collection) were cultured in DMEM (Dulbecco's modified Eagle's medium) supplemented with glutamine, penicillin, streptomycin and 10% (v/v) calf serum (Invitrogen).

### Cytokine production

BMDC (bone marrow-derived dendritic cell; 7×10^4^ cells) were incubated for 15 min in 96-well round bottom plates in complete RPMI. Cells were either left untreated or were pre-treated with DMSO, 2 μM PD184352 [provided by the DSTT (Division of Signal Transduction Therapy), University of Dundee] or 0.1 μM BIRB0796 (DSTT) for 30 min at 37°C before being stimulated with 50 ng/ml LPS (Axxora) for 18 h at 37°C. In some experiments, BMDC were pre-treated with various concentrations of D1870 or BIX (kind gifts of Boehringer-Ingelheim Pharmaceuticals) for 30 min or 1 h 30 respectively at 37°C. The amounts of TNFα (tumour necrosis factor α), IL6 (interleukin 6) and IL10 in the supernatant were measured using ELISA kits specific for each cytokine (TNFα, IL6; Peprotech, IL10; R&D Systems).

### Dextran uptake

FITC-dextran uptake was measured as described [19]. Briefly, BMDC (2×10^5^ cells) plated in 96-well plate in complete RPMI were either untreated or treated with various amount of D1870 or BIX for 30 min or 1h30 at 37°C, respectively. In some experiments, cells were pre-treated with 1 μM Akt-i (Merck) for 30 min. Cells were left unstimulated or were stimulated with 50 ng/ml LPS for 20 min at 37°C followed by the addition of 1 mg/ml FITC dextran (Invitrogen) for 10 min at 37°C. Cells were washed four times at 4°C in PBS supplemented with 2% (v/v) FCS (foetal calf serum) and then stained with APC (allophycocyanin)-labelled anti-CD11c antibody (BD biosciences). FITC dextran uptake was measured on an FACS Calibur (BD biosciences).

### Cell stimulation and cell lysate preparation

BMDC (1.5×10^6^ cells) were incubated in 6-well plates in RPMI supplemented with 0.3% FCS for 5 h at 37°C. Cells were pre-treated with various amounts of D1870 or BIX for 30 min or 1h30 at 37°C followed by stimulation with 50 ng/ml LPS for 30 min or with 100 ng/ml MIP1α (macrophage inflammatory protein 1α; Peprotech) for 5 min at 37°C. NIH3T3 cells (2×10^4^) were plated in 6-well plates for 48 h at 37°C. The cells were deprived of serum for 8 h in DMEM supplemented with 2 mg/ml BSA (Sigma) and then stimulated with 100 ng/ml EGF (epidermal growth factor; Peprotech) for 5 min at 37°C.

Cells were washed in cold PBS, lysed on ice for 10 min in lysis buffer (1% Triton X-100 containing 50 mM Tris–HCl (pH 7.5), 150 mM NaCl, 1 mM EGTA, 1 mM EDTA, 10 mM NaF, 1 mM sodium orthovanadate and 5 mM sodium pyrophosphate and protease inhibitors (Roche)) and centrifuged for 10 min at 20000 ***g*** at 4°C. Equal amounts of protein were separated by electrophoresis on 4–12% NuPage gels (Invitrogen) and then transferred onto nitrocellulose membranes (Amersham). Membranes were probed with the following antibodies against: p-FLNa (Ser^2125^), p-Bad (Ser^112^), p-GSK3 (glycogen synthase kinase 3) α/β (Ser^21^/Ser^9^), p-Erk1/2, p-p38, p-MK2 (Thr^334^), p-Rsk (Ser^386^), p-JNK (c-Jun N-terminal kinase), p-IκBα (Ser^32^), p-MSK1 (Ser^376^), p-PKB (protein kinase B, Ser^473^) and p-PKB (Thr^308^), total PKB, p-ERM (ezrin, radixin and moesin; Thr^567^/Thr^564^/Thr^558^), total ERM all from Cell Signalling; p-Rsk (Ser^227^) from R&D Systems and Rsk2 (Santa Cruz Biotechnology).

### Immunoprecipitation of paxillin

BMDC (5×10^6^ cells) were stimulated as described above. Lysates were pre-cleared with protein G Sepharose beads (Roche) for 30 min at 4°C. Paxillin was immunoprecipitated with 2 μg of anti-paxillin (Millipore) or with anti-IgG antibodies (Santa Cruz Biotechnology) for 1 h at 4°C and then incubated with 15 μl protein G Sepharose beads for 1 h at 4°C. Beads were washed four times with lysis buffer and paxillin was eluted with NuPage sample buffer (Invitrogen). The immunoprecipitated proteins were separated by electrophoresis on a 10% (w/v) NuPage gel and transferred onto a nitrocellulose membrane. The membrane was probed with anti-phospho Ser250-paxilin (kind gift of Luc Sabourin, McMaster University, Canada) or anti-paxillin antibodies.

### SDC (spleen dendritic cells) labelling in SILAC media and enrichment of ATP-binding proteins

SDC were cultured for at least 14 days in RPMI (without arginine and lysine, custom-made by Biosera) containing 10% dialysed FCS (Hyclone) supplemented with 84 mg/l of L-arginine (Sigma) and 40 mg/l L-lysine (Sigma) for the ‘light’ medium or 84 mg/l of L-arginine ^13^C_6_ or ^13^C_10_ and 40 mg/l L-lysine ^13^C_6_ (Cambridge Isotope Laboratory) for the ‘heavy’ medium. Equal numbers (2×10^7^) of ‘light’ or ‘heavy’ labelled SDCs were incubated in RPMI containing 0.5% FCS for 3 h in 15 cm dishes. The cells were lysed in the lysis buffer provided in the kit (Pierce). Equal amounts (1 mg for Western blot, 500 μg for mass spectrometry analysis) of desalted cell lysates were untreated (light) or pre-treated with 10 μM D1870 (light or heavy) or 10 μM BIX (heavy) for 20 min at 20°C. Labelling with the desthiobiotin ATP probe, and precipitation with streptavidin beads were performed according to the manufacturer's instructions (Pierce kinase enrichment kit or ActivX™). Equal amount of streptavidin precipitates were separated by electrophoresis as described above and probed with antibodies against Rsk2, Lok (Bethyl), Slk (Bethyl), PLK1 (Calbiochem), p38, Erk2 and Mst1 (from Cell Signaling).

### Mass spectrometry and data analysis

Desthiobiotin-labelled tryptic peptides were precipitated using streptavidin beads. The peptides were eluted using 50% acetonitrile/0.1% tri-fluoroacetic acid then equal volumes of light and heavy labelled peptides were mixed and lyophilized. Quantitative mass spectrometry analyses were performed essentially as previously described [22]. Protein digests were resuspended in 0.1% (v/v) FA (formic acid) and injected onto a 2 cm × 100 μm trap column and separated on a 15 cm × 75 μm Pepmap C18 reversed-phase column (Thermo Fisher Scientific) on a Dionex 3000 Ultimate RSLC. Peptides were eluted by a linear 60 min gradient (95 min total run) of 95% A/5% B to 35% B (A: H_2_O, 0.1% FA; B: 80% ACN, 0.08% FA) at 300 nl/min into a LTQ Orbitrap Velos (Thermo Fisher Scientific). Data were acquired using a data-dependent ‘top 15’ method, dynamically choosing the most abundant precursor ions from the survey scan (335–1800 Th, 60000 resolution, target value 10^6^). Precursors above the threshold of 5000 counts were isolated within a 2 Th window and fragmented by CID in the LTQ Velos using normalized collision energy of 35 and an activation time of 10 ms. Dynamic exclusion was defined by a list size of 500 features and exclusion duration of 45 s. Unassigned charge states and charge state 1 were rejected. Lock mass was used and set to 445.120024 for ions of PCM (polydimethylcyclosiloxane).

SILAC quantitation was performed using MaxQuant v1.3.0.5 [23]. Mass spectrometric runs of four biological replicates of D1870 versus untreated, BIX versus untreated and BIX versus D1870 were searched against a combined *Mus musculus* Uniprot-Trembl database (as of 18.10.2012) containing 50543 sequences and a list of common contaminants in proteomics experiments (247 entries). The following settings were used: enzyme trypsin, allowing for one missed cleavage, fixed modifications were carbamidomethyl (C), variable modifications were set to Desthiobiotin (K), Acetyl (Protein N-term) and Oxidation (M). MS/MS tolerance was set to 0.5 Da, precursor tolerance was set to 6 ppm. Peptide and Protein FDR (estimated by searching against the reversed database) was set to 0.01, minimal peptide length was 7, and one unique peptide was required. Peptide/protein ratios were obtained from the DesthiobiotinSites.txt file and positively quantified samples required calculated ratios out of two of the four replicates. *P*-values were determined by Student's *t* test.

### Kinase assay for recombinant Rsk2, Mst1, Lok and Slk

Recombinant Rsk2 (12 ng/reaction), GST-Mst1 (35 ng/reaction), GST-Lok (375 ng/reaction) (all from DSTT) and rSlk (200 ng/reaction) (Millipore), diluted in 50 mM Tris–HCl (pH7.5), 0.1 mM EGTA, were treated with various amounts of D1870 or BIX for 10 min at 20°C and then added to a reaction mix containing 50 mM Tris–HCl pH7.5, 0.1 mM EGTA, 100 μM γ^32^P-ATP (25 μM for Lok and Slk) 10 mM MgCl_2_, 2 mM DTT and the substrates; 30 μM CROSSTIDE (GRPRTSSFAEG) for Rsk2, 0.33 mg/ml myelin basic protein for Mst1, 100 μM AXLTIDE (KKSRGDYMTMQIG) for Lok and 1.25 mg/ml bovine histone H1 for Slk. The kinase reaction for Lok and Slk was supplemented with 12.5 mM sodium glycerophosphate and 0.25 mM sodium orthovanadate. The reactions were incubated for 15–20 min at 30°C then terminated and analysed as described previously [14].

### Measurement of Mst1, Lok and Slk activities in BMDC

Equal numbers of BMDC (1×10^7^/dish) were incubated in 15 cm dishes in RPMI supplemented with 0.3% FCS for 3 h at 37°C then incubated for 30 min with or without 50 ng/ml LPS. The cells were washed once in cold PBS and lysed in the lysis buffer described above. Mst1, Lok and Slk were immunoprecipated from 150 μg, 850 μg and 1 mg of cell lysates respectively with 2 μg of anti-MST1 (Cell Signalling), anti-Slk (Bethyl) or anti-Lok (Bethyl) or control IgG (Santa Cruz Biotech) coupled to G-protein Sepharose (Amersham) for 1 h at 4°C. The beads were washed once with lysis buffer, once with lysis buffer containing 0.5 M NaCl followed by two washes with kinase buffer (50 mM Tris–HCl (pH7.5), 0.1 mM EGTA, 1 mM DTT, 12.5 mM sodium glycerophosphate, 0.25 mM sodium orthovanadate). Kinase assays were performed as described above.

### Statistical analysis

Statistical significance was assessed by one way ANOVA. Differences with *P* values of <0.05 were considered statistically significant.

## RESULTS

### Rsk inhibitors D1870 and BIX differentially affect cytokine production

**Figure 1:**
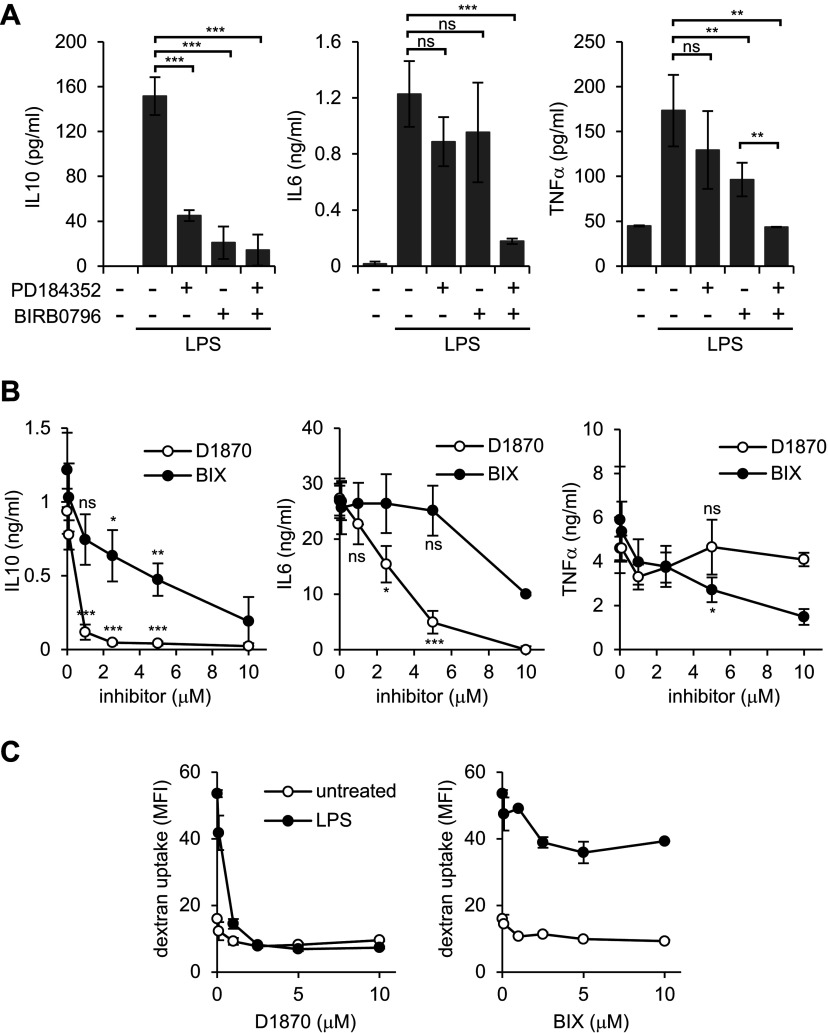
D1870 and BIX affect differentially cytokine production and macropinocytosis in DC (**A**) IL6, IL10 and TNFα production by BMDC stimulated for 18 h with LPS (50 ng/ml) in the absence or in the presence of MEK inhibitor, PD184523 (2 μM) or p38 inhibitor, BIRB0796 (0.1 μM). Data are the mean±S.D. of triplicate stimulations and are representative of three independent experiments. (**B**) IL6, IL10 and TNFα production by BMDC stimulated for 18 h with LPS (50 ng/ml) in the presence of increasing concentrations of D1870 or BIX. Data are the mean±S.D. of triplicate stimulations and are representative of three independent experiments. (**C**) Uptake of FITC-dextran (1 mg/ml) by DCs left untreated or treated with LPS for 30 min in the presence of increasing concentrations of D1870 (left) or BIX (right). The median intensity fluorescence is shown. Data are the mean±S.D. of triplicate stimulations and are representative of three independent experiments.*P<0.05, **P<0.01, ***P<0.001, ns not significant.

Several studies have shown that activation of Erk1/2 and p38 is crucial for the production of several pro- and anti-inflammatory cytokines in DC upon TLR stimulation [24,25]. These data were obtained from DC deficient either in p38 isoforms or in tpl2, an upstream activator of Erk1/2 or by using inhibitors of the p38 and Erk1/2 pathways. Indeed, in BMDC stimulated with LPS in the presence of both the MEK1/2 inhibitor PD184352 and the p38 inhibitor BIRB0796, the production of TNFα, IL6 and IL10 was blocked (Figure 1A). Because Rsk is activated by both Erk1/2 and p38 in DC [19], we asked if Rsk could regulate cytokine production downstream of p38 and Erk1/2.

To address this we stimulated BMDC with LPS in the absence or presence of increasing concentration of two different Rsk inhibitors: D1870 [13] and BIX [15]. Surprisingly, whereas D1870 resulted in a dose-dependent inhibition of IL6 and IL10 secretion, BIX only partially blocked IL10 and IL6 production (Figure 1B). In contrast, TNFα production was partially blocked by BIX but not by D1870 (Figure 1B). Analysis of the mRNA levels of IL6 and IL10 after 1 or 3 h of LPS stimulation in the presence of D1870 showed that the inhibitor rapidly inhibited the production of mRNA for these cytokines (data not shown). This discrepancy in the results obtained with the two inhibitors prompted us to revisit the effect of Rsk inhibitors on TLR-mediated macropinocytosis. As shown previously [19], D1870 completely blocked LPS-mediated dextran uptake (Figure 1C). In contrast, increasing concentrations of BIX blocked TLR stimulated macropinocytosis by only ~20% (Figure 1C).

### PKB activation is specifically blocked in DC by the D1870 inhibitor

**Figure 2:**
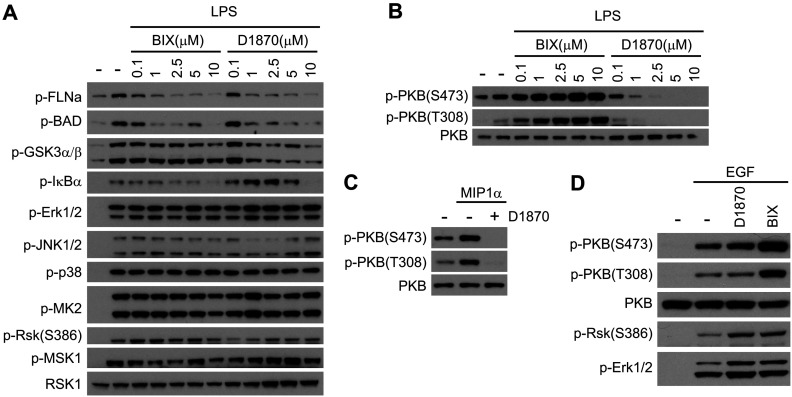
D1870 selectively blocks PKB phosphorylation in DC (**A–B**) BMDC were stimulated for 30 min with LPS (50 ng/ml) in the presence of increasing concentrations of D1870 or BIX. (**C**) DCs were stimulated for 5 min with MIP1α (100 ng/ml) in the absence or in the presence of 4 μM D1870. (**D**) NIH3T3 cells were stimulated for 10 min with EGF (100 ng/ml) in the absence or in the presence of 4 μM D1870 or 2 μM BIX. Data are representative of three independent experiments.

The above data suggested that D1870 not only blocked Rsk but was likely having other effects, which perturbed TLR signalling in DC. These potential off-target effects could explain the differences observed between D1870 and BIX. Although both compounds were previously shown to block Rsk kinases at low nM concentrations *in vitro* (see also Figure 5A) we wanted to establish that Rsk activity in DC was fully and equivalently inhibited by both compounds. We monitored the phosphorylation of several well-known Rsk substrates in BMDC including filamin A on Ser^2152^ [26], GSK3β on Ser^9^ [27] and Bad on Ser^112^ [28] in the presence of increasing amounts of either BIX or D1870 (Figure 2A). LPS signalling stimulated the phosphorylation of filamin A and Bad and both inhibitors blocked the phosphorylation of these substrates at concentrations as low as 1 μM (Figure 2A). Although both compounds blocked GSK3α/β phosphorylation, D1870 inhibition was more potent. These results indicate that the two inhibitors are effective in blocking Rsk activation in cells. Next, we investigated their effect on the three main pathways known to regulate cytokine production and/or macropinocytosis namely the MAPK [17,24], the NFκB [29] and the PI3K (phosphoinositide-3 kinase) [30,31] signalling cascades. Neither inhibitor had any effect on Erk1/2, p38 or JNK1/2 phosphorylation (Figure 2A). Phosphorylation of MSK1 and MK2, two additional downstream substrates of Erk1/2 and/or p38, were also not affected confirming previous data in other cell types [13]. Although it has been shown that in tumour cell lines IκBα phosphorylation is mediated by Rsk [32], this appeared not to be the case in DC since, neither D1870 nor BIX affected the phosphorylation of IκBα (Figure 2A).

Next we analysed the phosphorylation of a PI3K downstream effector: PKB. To our surprise, LPS-mediated PKB activation was oppositely affected by the two compounds. Whereas D1870 treatment resulted in the complete block of PKB phosphorylation at both its activator sites Ser^473^ [33] and Thr^308^ [34], BIX increased the phosphorylation of both sites (Figure 2B). A similar effect of D1870 was observed on PKB phosphorylation in insulin stimulated 3T3-L1 adipocytes [45]. To see whether the D1870 effect on PKB phosphorylation was stimulus dependent, we treated DC with the chemokine MIP1α in the presence of the D1870 inhibitor (Figure 2C). Again, PKB phosphorylation was impaired showing that D1870 blocked the activation of PKB in DC independently of the stimulus. However, in agreement with an earlier report [13], when we stimulated the fibroblast cell line NIH3T3 with EGF in the presence of D1870 PKB phosphorylation was not affected (Figure 2D). Taken together these results demonstrate cell-type-specific inhibitory effects of D1870 on the PKB pathway. On the other hand, the increase in PKB activation seen with BIX is consistent, although more dramatic, with that seen previously in skeletal muscle from Rsk2-deficient mice [35].

### Combining BIX with a PKB inhibitor does not recapitulate D1870 effects in DC

**Figure 3:**
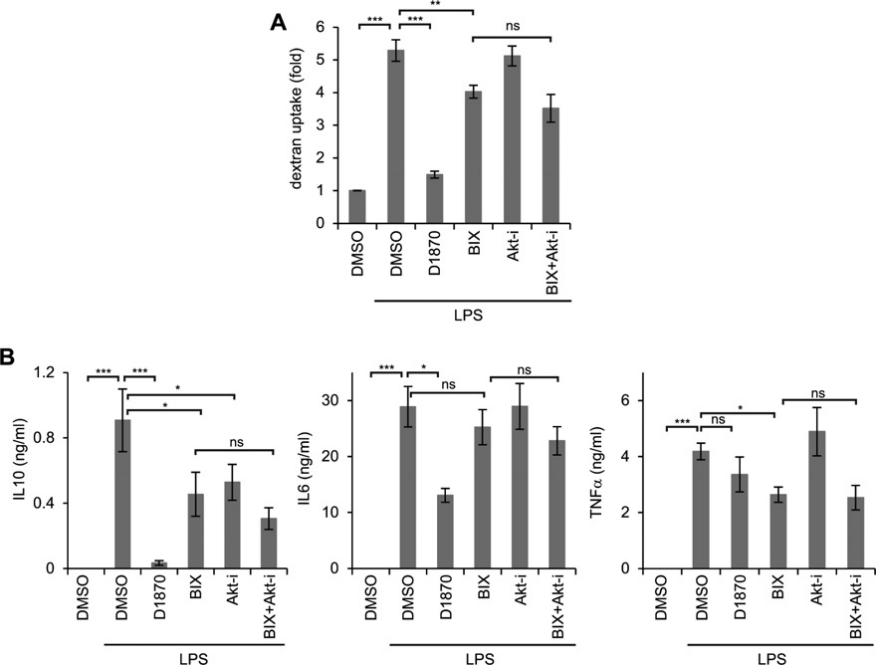
Combination of BIX and PKB inhibitor Akt-i does not recapitulate D1870 effect on cytokine production and macropinocytosis (**A**) Uptake of FITC-dextran (1 mg/ml) by DCs left untreated or treated with LPS (50 ng/ml) for 30 min in the presence of D1870 (4 μM), BIX (2 μM) or Akt-i (1 μM). Results are presented as fold induction (median intensity values) relative to uptake by unstimulated cells. Data are the mean±S.D. of duplicate stimulations and are representative of three independent experiments. (**B**) IL6, IL10 and TNFα production by BMDC stimulated for 18 h with LPS in the absence or in the presence of D1870 (4 μM), BIX (2 μM) or Akt-i (1 μM). Data are the mean±S.D. of triplicate stimulations and are representative of three independent experiments.*P<0.05, **P<0.01, ***P<0.001, ns not significant.

Because PKB isoforms have been implicated in the regulation of pro-inflammatory cytokine production in macrophages [36] and in macropinocytosis in Dictyostelium [37] it seemed possible that the effects of D1870 in DC might be explained by the combined inhibition of Rsk and PKB. To test this we used Akt-i [38] a highly specific inhibitor of PKBα and β [14]. We measured dextran uptake in DC stimulated with LPS in the presence of BIX and Akt-i alone or in combination. The results showed that blocking PKB alone had no effect on dextran uptake (Figure 3A). Moreover, the combination of both BIX and Akt-i did not further block macropinocytosis. Similarly, blocking PKB had no additional effect on cytokine production by LPS-stimulated DC (Figure 3B). Therefore although a potential involvement of PKB cannot be excluded, the combined inhibition of Rsk and PKB is not sufficient to recapitulate the effect of D1870 pointing to alternative off-target effects of this inhibitor. Sensitivity to D1870 has been reported for some other kinases, notably PLK1 (IC_50_ 100 nM) and Aurora B (IC_50_ 340 nM) although to a weaker extent compare with Rsks (IC_50_ 10–30 nM) [13,14]. We treated BMDC with either BI2536 [39] or ZM447439 [40], PLK and aurora inhibitors respectively, or in combination with BIX and Akt-i. None of the combinations tested were able to recapitulate the effect of D1870 (data not shown) suggesting that D1870 is blocking one or more additional kinases.

### Nucleotide acyl phosphate labelling and SILAC to identify inhibitor targets

**Figure 4:**
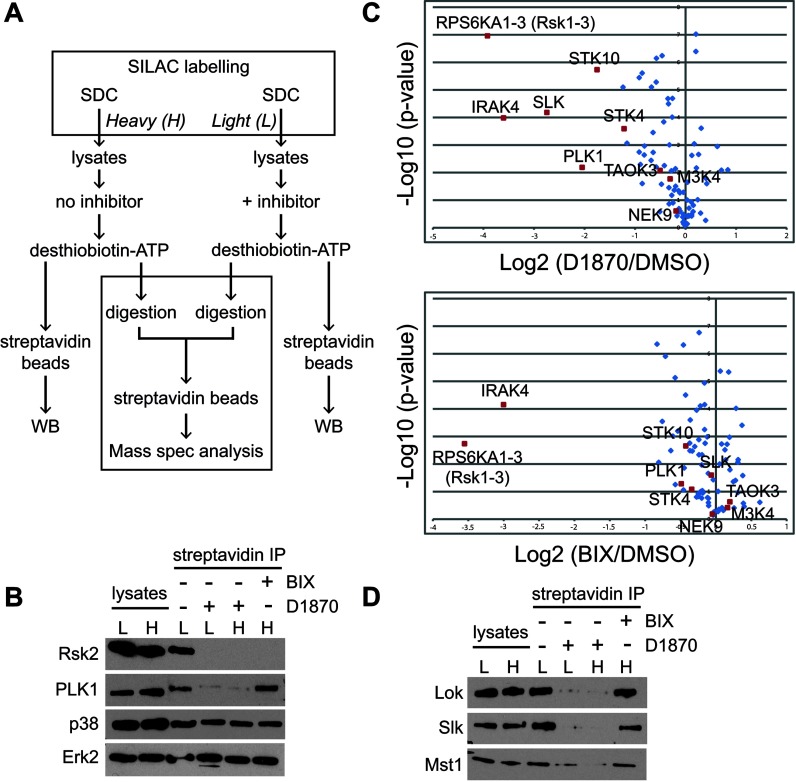
Identification of D1870 targets by nucleotide acyl phosphate labelling (**A**) Diagram of the work flow (**B**, **D**) Rsk2, PLK1, Lok, Slk, Mst1, p38 and Erk2 amounts in streptavidin precipitates from nucleotide acyl phosphate-labelled DC lysates. Data are the mean of four independent experiments. (**C**) Volcano plot shows the Log2 D1870/DMSO ratio (upper panel) or Log2 BIX/DMSO ratio (lower panel) against the -Log10 of the *P* value for four independent experiments.

The fact that D1870 and BIX differentially impaired several important DC responses to TLR-signalling prompted us to try to identify additional enzymes targeted by D1870, but not by BIX. There are ~500 kinases encoded by the mouse genome and the available panels for drug screening are so far limited to up to 250 kinases. In addition, D1870 being an ATP competitor could potentially affect other ATP-utilizing enzymes. To increase our chance of identifying additional D1870 targets we adopted a strategy, which in principle would allow us to identify such targets directly in DC lysates. We used two technologies to aid the side-by-side analysis of the two Rsk inhibitors. Firstly, to reveal inhibitor targets we used desthiobiotinylated acyl phosphate nucleotides to covalently tag ATP utilizing enzymes in DC lysates [21]. Secondly, to facilitate pairwise analysis of complex mixtures of desthiobiotinylated modified enzymes we cultured DC in the SILAC medium using either light or heavy amino acids. The former technology exploits a conserved lysine residue neighbouring the active site of kinases and other ATP-utilizing enzymes, which covalently reacts with the acyl phosphate bond in enzyme-bound desthiobiotin-ATP or ADP resulting in covalent transfer of desthiobiotin to the enzyme. Tryptic digestion and purification of desthiobiotin-modified peptides on streptavidin beads allows identification of successfully modified enzymes by mass spectrometry. Inclusion of an active site reactive inhibitor prevents reaction with the desthiobiotin-nucleotide and therefore the recovery of the relevant peptide from that enzyme [21]. Nucleotide acyl phosphates were shown to react with ~80% of known kinases including different Rsk isoforms and the known additional D1870 targets PLK1 and Aurora [21]. It seemed reasonable therefore to assume that additional D1870 targets would also engage with and become modified by these nucleotides. Lysates from DC grown in light (L-DC) or heavy (H-DC) SILAC media were labelled with desthiobiotin-ATP in the presence (L-DC) or absence (H-DC) of Rsk inhibitors as described in the Materials and methods section. Labelled proteins were either recovered directly on streptavidin beads for Western blot analysis or subjected to digestion with trypsin to release biotin-labelled active site peptides, which were then combined and subjected to quantitative liquid chromatography (LC) MS analysis (Figure 4A). As expected, diverse protein kinases and other probe-reactive proteins were recovered on streptavidin beads following desthiobiotin-ATP labelling of DC lysates including Rsk2, Erk1/2 and p38. Moreover, the capture of Rsk2 but not Erk1/2 or p38, was blocked by the inclusion of either D1870 or BIX confirming specific active site blocking by the two inhibitors (Figure 4B). Recovery of PLK1 was not affected by the presence of BIX but was blocked by D1870 confirming previous screening data, which identified PLK1 as a D1870 target [14]. This result demonstrates the potential of the methodology to identify additional D1870 targets. To identify such targets we performed quantitative SILAC-based, high-resolution mass spectrometry experiments (four biological replicates) of D1870-treated samples (H) against DMSO treated (control, L), BIX (H) against control (L) and BIX (H) against D1870 (L). Altogether, we identified 2216 desthiobiotin-labelled peptides of which 886 were reproducibly quantified in two out of four replicates (Supplementary Table S1 available at http://www.bioscirep.org/bsr/034/bsr034e091add.htm). In D1870-treated samples, Rsk exhibited the lowest ratio consistent with expectations and PLK1 also showed an H/L ratio smaller than 1 (Table 1 and Supplementary Table S1). However, we consistently observed H/L ratios <1 for several additional kinases including IRAK4, Slk, Lok (STK10) and Mst1 (STK4) indicating that D1870 suppressed the desthiobiotin modification of their active sites. These data are most easily represented in the ‘volcano’ plot shown in Figure 4(C), which shows the Log2 ratio (D1870/DMSO) against the -Log10 of the *P* value for the four independent experiments. When we substituted D1870 with BIX and repeated the experiments only IRAK4 (and Rsk2) deviated significantly from a ratio of 1 (Figure 4C). Thus, the reaction of Slk, Lok and Mst1 with the desthiobiotin probe was suppressed by D1870 but to a much lesser extent, if at all, by BIX. Importantly, direct Western blotting of DC lysates confirmed the differential sensitivity of Slk, Lok and Mst1 to the two Rsk inhibitors (Figure 4D). Interestingly, all three kinases are found in the STE-20 like kinase family of the Kinome tree [41].

**Table 1:** Selected identified desthiobiotin peptides

Identifiers	Protein name	BIX/D1870	*P*-value	BIX/DMSO	*P*-value	D1870/DMSO	*P*-value	Score	Sequence and desthiobiotin probabilities
KS6A3_MOUSE KS6A2_MOUSE KS6A1_MOUSE	Ribosomal protein S6 kinase alpha 1/2/3	0.99	6.6E-01	0.09	1.8E-03	0.07	1.1E-07	158	DLK(1)PENILLDEEGHIK
IRAK4_MOUSE	IL-1 receptor-associated kinase 4	0.81	2.6E-01	0.12	7.0E-05	0.08	1.0E-04	303	DIK(1)SANILLDKDFTAK
SLK_MOUSE	STE20-like serine/threonine-protein kinase	2.10	1.4E-02	0.96	2.5E-02	0.15	6.6E-05	214	DLK(1)AGNILFTLDGDIK
PLK1_MOUSE	Serine/threonine-protein kinase PLK1	1.99	1.0E-03	0.71	5.2E-02	0.24	6.5E-03	153	EVFAGK(1)IVPK
STK10_MOUSE	Serine/threonine-protein kinase 10	2.05	1.9E-04	0.75	2.2E-03	0.30	1.8E-06	266	DLK(1)AGNVLMTLEGDIR
STK4_MOUSE	Serine/threonine-protein kinase 4	1.89	1.4E-02	0.79	8.3E-02	0.43	2.6E-04	146	ETGQIVAIK(1)QVPVESDLQEIIK
TAOK3_MOUSE	Serine/threonine-protein kinase TAO3	1.79	2.8E-05	1.15	2.3E-01	0.71	8.3E-03	223	FATIK(1)SASLVTR
M3K4_MOUSE	Mitogen-activated protein kinase kinase kinase 4	1.87	9.5E-04	1.12	3.8E-01	0.81	1.7E-02	227	DIK(1)GANIFLTSSGLIK
NEK9_MOUSE	Serine/threonine-protein kinase Nek9	1.73	2.6E-03	0.97	6.5E-01	0.88	2.5E-01	130	DIK(1)TLNIFLTK

### Differential inhibition of Slk, Lok and Mst1 by D1870 versus BIX

**Figure 5:**
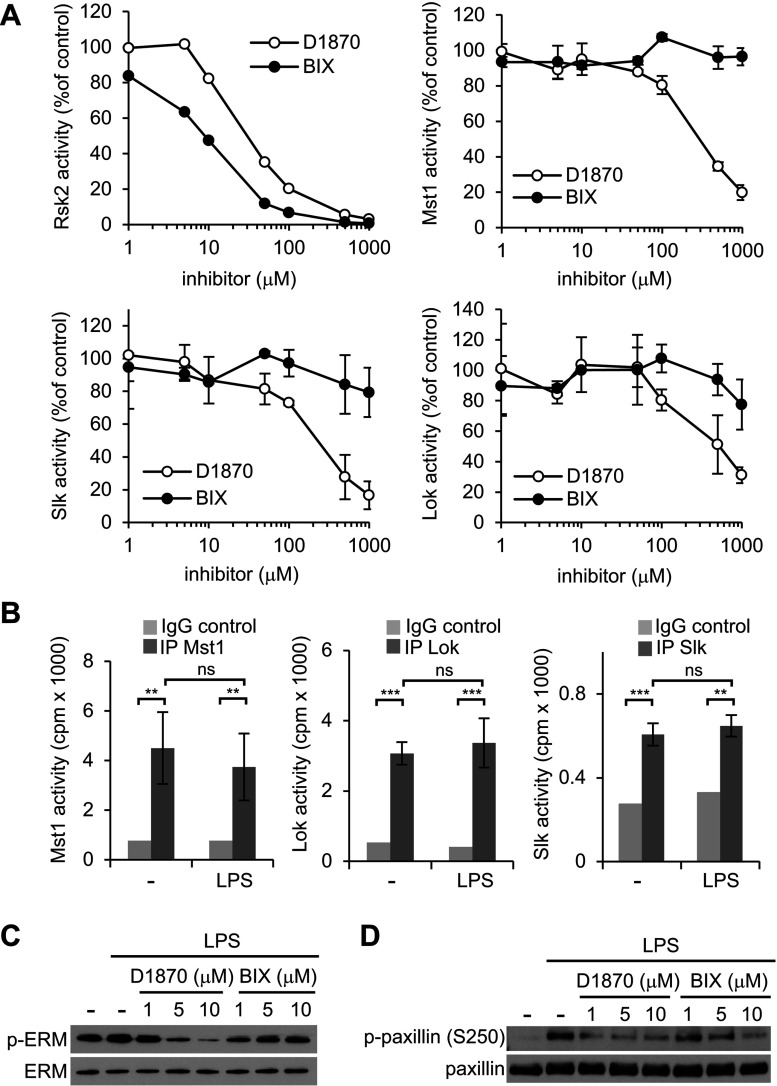
Slk, Lok and Mst1 are inhibited by D1870 *in vitro* and in living cells Activated Rsk2, Mst1, Lok and Slk were assayed in the absence or presence of increasing concentrations of D1870 or BIX02565 as indicated in the materials and methods section. The results are presented as percentage of kinase activity relative to the control measured in absence of the inhibitors. Data are the mean±S.D. of duplicate experiments. (**B**) Mst1, Lok and Slk activity in DC either unstimulated or stimulated for 30 min with LPS (50 ng/ml). The background activity was determined using non-specific IgG for the immunoprecipitation. Data are the mean±S.D. of triplicate stimulations and are representative of three independent experiments. *P<0.05, **P<0.01, ***P<0.001, ns not significant. (**C–D**) Phosphorylation of ERM (**C**) and paxillin (**B**) was assayed by immunoblotting of lysates from DC stimulated for 30 min with LPS in absence or in the presence of D1870 or BIX at the indicated concentrations. Data are representatives of three independent experiments.

Slk, Lok and Mst1 were not included in the panel of enzymes originally used to test the specificity of D1870 although Mst2 was present and indeed showed some sensitivity to this inhibitor [14]. We therefore investigated directly the ability of the two compounds to inhibit the protein kinases identified by nucleotide acyl phosphate labelling technology. As shown in Figure 5(A), recombinant Rsk2 was blocked by increasing concentrations of both D1870 and BIX with IC_50_ of 35 and 9 nM, respectively, in broad agreement with previous data [13,15]. In contrast, recombinant Slk, Lok and Mst1 were inhibited by D1870 but not by BIX. For Mst1 the IC_50_ was ~300 nM, whereas for Slk and Lok the respective IC_50_ were 250 and 500 nM. Next, we assessed whether Slk, Lok and Mst1 were active in DC. When Lok and Mst1 were immunoprecipitated from DC and tested *in vitro*, kinase activity was detected, which was substantially inhibited by D1870. Compare with Lok and Mst1, the activity measured for Slk was less strong but it was significantly blocked by D1870. Moreover, we recovered activity from both resting and LPS-stimulated DC indicating that these enzymes are constitutively active in DC (Figure 5B and Supplementary Figure S1 available at http://www.bioscirep.org/bsr/034/bsr034e091add.htm).

The above results suggest that some of the documented effects of TLR signalling in murine DC, for example, reorganization of the actin cytoskeleton, may in fact be driven by one or more members of the Ste-20 kinase family. In fact, several studies have shown that Slk and Lok regulate actin cytoskeleton dynamics. For instance, Lok regulates the migration and polarization of T cells [42], whereas Slk controls the turnover of focal adhesion in fibroblasts through phosphorylation of paxillin [43]. Both kinases have been implicated in the phosphorylation of ezrin, a member of the ERM protein family, which links membrane proteins to the actin cytoskeleton [44]. We therefore asked whether the phosphorylation status of ERM proteins in DC was affected by the Rsk inhibitors. As shown in Figure 5(C), ezrin, moesin and radixin were constitutively phosphorylated in DC and therefore maintained in their active state in the absence of LPS stimulation most likely due to the basal activity of Lok and/or Slk. Activation of DC by LPS resulted in a small increase in their phosphorylation. However, treatment with D1870 but not with BIX resulted in a substantial decrease in the phosphorylation of ERM proteins, the phosphorylation of ezrin and/or radixin being the most affected (Figure 5C). We also looked at the phosphorylation of paxillin on Ser^250^, which was recently shown to be an Slk target [43]. Conversely to the ERM proteins, paxillin phosphorylation at Ser^250^ was not detectable in resting DC but was observed upon LPS stimulation. As shown in Figure 5(D) D1870 prevented its phosphorylation at low concentrations whereas BIX had little effect. Taken together these results strongly suggest that D1870 inhibits Lok and Slk in living cells and suppresses phosphorylation of key cytoskeletal substrates. Consistent with this, D1870 induced morphological changes in resting DC, which included enhanced cell spreading, more pronounced focal adhesion and a partial loss of actin-rich podosomes. These changes were not observed upon BIX treatment (Supplementary Figure S2 http://www.bioscirep.org/bsr/034/bsr034e091add.htm).

## DISCUSSION

The Rsk kinases have emerged as important downstream effectors of the mitogen- and stress-activated kinases Erk1/2 and p38. As recently reviewed by Romeo et al. [1] Rsk has been implicated in transcriptional regulation, cell cycle progression, protein synthesis, cell growth, cell survival and cell migration. The presence of multiple isoforms makes their genetic ablation more difficult and to date only Rsk2-deficient mice have been investigated. Several cell permeable inhibitors have been developed including D1870, which binds reversibly to the N-terminal kinase domain of all Rsk isoforms. D1870 was extensively characterized in an earlier study and shown to be specific for Rsk across a large panel of kinases tested including other members of the AGC family [13]. Moreover, in cellular assays activation of PKB, PKA and other kinases by growth factors and other stimuli was similarly unaffected by D1870. Here, we extended our earlier study that identified a role for Rsk in DC biology [19]. We compared D1870 with a more recently described and structurally different Rsk inhibitor BIX, which also blocks all four Rsk isoforms. We confirmed that LPS-mediated macropinocytosis and cytokine production were dependent on Rsk activity. However, we found that inhibition of these processes varied substantially depending on the inhibitor used, D1870 having the strongest effect. Using a method to probe potential inhibitor targets directly in cell lysates, we showed that D1870 blocked not only Rsk but also several members of the Ste20-like kinase family, namely Slk, Lok and Mst1. Our results also show cell-type-specific inhibitory effects of D1870 on other kinase pathways, notably those driving PKB activation.

Indeed, conversely to the situation in NIH3T3 or HEK-293 (human embryonic kidney cells) cells, in DC D1870 blocked the activation of PKB. Chen et al. showed a similar effect of D1870 on PKB phosphorylation in insulin stimulated 3T3-L1 adipocytes [45], although the effect was not as strong as in DC. How D1870 affects PKB phosphorylation is not yet clear. PKB activation requires binding to the PI3K products PIP3 (phosphatidylinositol-3,4,5-triphosphate) via its PH domain, phosphorylation of Ser^473^ by rictor (mTORC2 complex) (and potentially other kinases such DNA KA), followed by the phosphorylation of Thr^308^ by PDK1. One possibility is that D1870 suppresses the activity of a p110 PI3K isoform required for PKB activation in DC but not in fibroblasts. However, other mechanisms of bringing PDK1 and PKB into proximity, for example involving scaffolding proteins have been described [46]; so it is also possible that perturbation of the membrane proximal cytoskeleton via suppression of Slk and Lok activation might be responsible for D1870′s effects.

Surprisingly, BIX has the opposite effect on PKB: phosphorylation at both sites was increased. This is likely a genuine effect as PKB activation was also increased in muscle cells from Rsk2-deficient mice [35]. Moreover, preliminary data obtained in Rsk2-deficient DC stimulated with LPS showed that PKB phosphorylation is also enhanced (R. Zaru, unpublished work). This result indicates that Rsk negatively regulates PKB activation. The kinetics of Rsk and PKB activation by LPS in DC appear to be different. Rsk seems to reach its activation peak earlier compared with PKB (R. Zaru, unpublished work). It could be that Rsk, by preventing a premature PKB activation delays the termination of LPS-mediated signalling by some potential negative feedback triggered by PKB. This staggered activation of Rsk and PKB would also allow the sustained activation of the substrates that they have in common. For example, GSK3α/β has been reported to be phosphorylated not only by Rsk but also by PKB. This is likely what happens in DC as BIX only partially blocked GSK3 phosphorylation, whereas D1870 that blocks both Rsk and PKB had a much stronger effect. How Rsk mediates this feedback on PKB is at the moment unclear.

Further work will be needed to confirm how the Ste-20-like family kinases identified here contribute to TLR-signalled effects on the cytoskeleton in DC. However, all three have been linked to the regulation of the cytoskeleton in other cells and we were able to show that D1870 blocked the phosphorylation of ERM and paxillin, which are Lok and Slk substrates, respectively. These proteins have been found at the plasma membrane in T cells and fibroblasts and are well-known regulators of the actin cytoskeleton, which is required for macropinocytosis [47]. Although little is known about the role of ERM and paxillin in macropinocytosis, there is some evidence that could suggest their involvement. Indeed, ERM proteins, in their active state, are localized at the plasma membrane and in membrane ruffles, which in DC leads to the formation of macropinosomes. In addition, ERM proteins bind the Na^+^/H^+^ exchange protein NHE1, which is involved in the regulation of macropinocytosis in DC [48]. Although Mst1 has been characterized as a regulator of apoptosis [49], a study of DC lacking Mst1 showed that this kinase regulates also their adhesion and potentially their migration [50]. Whether the striking inhibitory effects of D1870 on cytokine production by DC could be due to targeting of Ste-20-like family kinases remains to be investigated.

Our data demonstrate that the D1870 compound should be used with caution particularly in immune and other cells where expression of Lok, Slk and Mst1 is prominent. Although the identification of novel D1870 targets has provided us with new clues on how to explain the dramatic effects that this compound has on DC functions it may be difficult to identified precisely the roles of each target as the effect observed could be the result of the combined inhibition of several of these kinases. At least for Rsk the study of deficient mice will help to clarify their role in DC biology alongside the data obtained with the other Rsk inhibitor BIX. Our study confirms the value of the nucleotide acyl phosphate labelling approach developed originally by Patricelli et al. [21]. It allows the target spectrum of an inhibitor with interesting physiological effects to be probed under conditions–i.e. the relative target levels and activity states–that are close to those found in the intact cell. Combining the approach with SILAC labelling and side-by-side analysis of inhibitors with the same nominal target but which have distinct physiological effects has revealed new targets and potentially new players in an important immune signalling system.

## Online data

Supplementary data
