# Supplementary material for: A combination of SILAC and nucleotide acyl phosphate labelling reveals unexpected targets of the Rsk inhibitor BI-D1870
Source: Biosci Rep. 2014 Jan 31;34(1):e00091. doi: 10.1042/BSR20130094 (PMC3908613; doi:10.1042/BSR20130094)
Supplement: Supplementary data [file bsr034e091add.pdf]

Biosci. Rep. (2014) / **34** / art:e00091 / doi 10.1042/BSR20130094

OPEN ACCESS

Original Paper

Bioscience Reports

www.bioscirep.org

Bioscience Reports

SUPPLEMENTARY DATA

A combination of SILAC and nucleotide acyl phosphate labelling reveals unexpected targets of the Rsk inhibitor BI-D1870

Alexander J. EDGAR*, Matthias TROST†, Colin WATTS*[1] and Rossana ZARU*[1]

*Division of Cell Signalling and Immunology, University of Dundee, Dow Street, Dundee DD1 5EH, U.K., and †MRC Protein Phosphorylation and Ubiquitylation Unit, College of Life Sciences, University of Dundee, Dow Street, Dundee DD1 5EH, U.K.

Supplementary Table S1 is available at http://www.bioscirep.org/bsr/034/bsr034e091add.htm

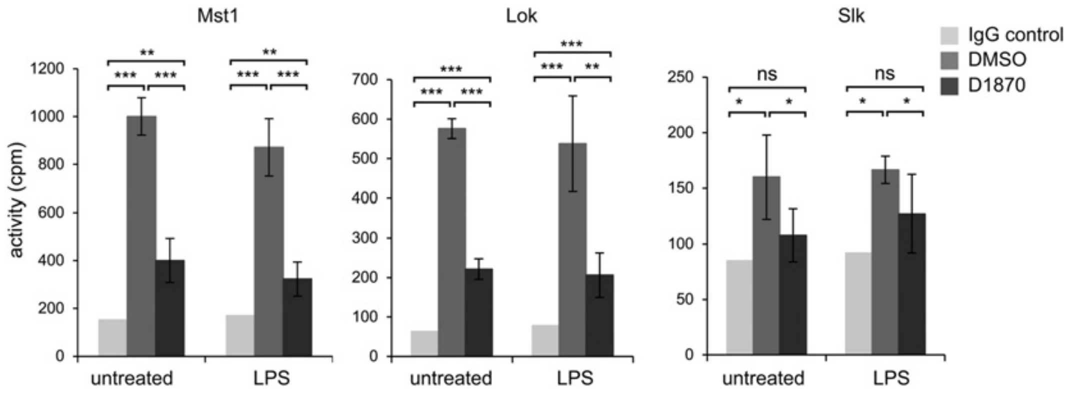

Figure S1 D1870 inhibitor blocks Mst1, Lok and Slk activities in DC lysates
Mst1, Lok and Slk activities in DC either unstimulated or stimulated with LPS (50 ng/ml) for 30 min at 37 °C. Immunoprecipitates were either untreated or treated with 1 μM D1870 prior measuring the kinase activity. Data are the mean±S.D. of triplicate stimulations. *$P < 0.05$, **$P < 0.01$, ***$P < 0.001$, ns not significant.

[1] Correspondence may be addressed to either of these authors (email c.watts@dundee.ac.uk or r.zaru@dundee.ac.uk).

© 2014 The Author(s) This is an Open Access article distributed under the terms of the Creative Commons Attribution Licence (CC-BY) (http://creativecommons.org/licenses/by/3.0/) which permits unrestricted use, distribution and reproduction in any medium, provided the original work is properly cited.

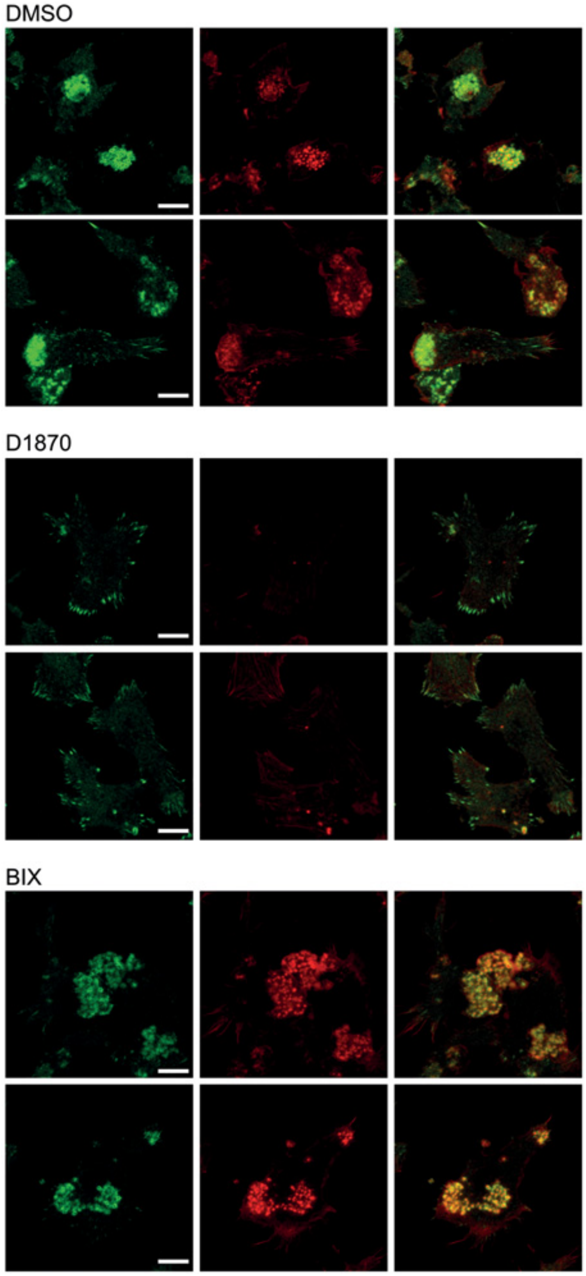

Figure S2 Actin cytoskeleton organisation is affected by D1870 inhibitor

DC (2.5×10^5), plated on coverslips, were treated with 4 μM D1870 or 2 μM BIX for 1h30 at 37 °C. Cells were fixed in 4% (v/v) paraformaldehyde for 15 min, permeabilized with 0.1% Triton for 5 min and stained with phalloidin-Alexa 555 (Invitrogen) and anti-vinculin antibody (Sigma) followed by goat anti-mouse Alexa 488 antibody (invitrogen). Cells were analysed on a Zeiss700 confocal microscope. Vinculin (green) and phalloidin (red). Error bar 10 μm. Images are representative of three independent experiments.

Received 9 September 2013/25 September 2013; accepted 10 October 2013

Published as Immediate Publication 17 December 2013, doi 10.1042/BSR20130094

© 2014 The Author(s) This is an Open Access article distributed under the terms of the Creative Commons Attribution Licence (CC-BY) (http://creativecommons.org/licenses/by/3.0/) which permits unrestricted use, distribution and reproduction in any medium, provided the original work is properly cited.
